# Feedback algorithms for intelligent reflecting surfaces: Phase shift matrix and single-bit feedback

**DOI:** 10.1371/journal.pone.0322183

**Published:** 2025-05-08

**Authors:** Sarmad Sohaib, Muhammad Ahmad, Muhammad Shafi

**Affiliations:** 1 Department of Electrical and Electronic Engineering, University of Jeddah, Jeddah, Saudi Arabia; 2 Department of Electrical Engineering, University of Engineering and Technology, Taxila, Pakistan; 3 School of Computing, Ulster University, Belfast, United Kingdom; Seoul National University of Science & Technology, KOREA, REPUBLIC OF

## Abstract

This paper introduces two innovative feedback algorithms—phase shift matrix (PSM)-based and single-bit feedback (SBF)-based—that improve the efficiency of phase adjustments in intelligent reflecting surfaces (IRS). In the PSM-based algorithm, the IRS phase shift vector is selected from a predefined PSM to maximize the output signal-to-noise ratio (SNR) at the receiver. The receiver then sends back only the index of the optimal phase shift vector to the transmitter. In contrast, the SBF algorithm adjusts the IRS elements using single-bit feedback. Here, the transmitter makes a minor random alteration in the phase of each IRS element at each iteration; simultaneously, the receiver transmits an SBF, indicating whether the SNR improved or deteriorated after the current iteration. The transmitter keeps the “good” phase adjustment and throws away the “bad” ones. Simulation results are produced to compare the performance of both algorithms in terms of average bit error rate, and results show that SBF-based phase adjustment of IRS elements is better than PSM-based phase adjustment.

## Introduction

The development of wireless communication systems has been traditionally aimed at optimizing transceivers to compensate for various channel impairments, which were mostly uncontrollable by system designers. The recent development of intelligent reflective surfaces (IRSs), empowered by advances in material sciences, has created a new paradigm in wireless communications. An IRS consists of many re-configurable reflective elements that are usually deployed on the exterior of obstacles/large buildings or cell edges. Reflected electromagnetic waves from different IRS elements can be combined constructively at the desired destination by intelligently adjusting the phase shifts of each IRS element, which ultimately increases the received signal-to-noise-power ratio (SNR) and coverage area [1–4]. Practically, an IRS behaves as a full duplex multiple-antenna amplify and forward relay with no self-interference and thermal noise added to the reflected signal. IRS is especially useful in dealing with some of the difficulties associated with high-frequency bands like millimeter waves, which suffer from significant path loss and limited penetration [5].

Although several objectives can be achieved using the IRS, many challenges are also involved in implementing an IRS-assisted wireless communication system [6, 7]. Among many open issues, in this paper, we only highlighted the problem of phase adjustment of IRS elements, which can be addressed by estimating channel state information (CSI) at the transmitter. There are two main issues in estimating CSI: (1) IRS elements cannot transmit/receive information, unlike relays, due to their passive nature, (2) and they are large in numbers. Only a few researchers have addressed this problem and provided their solutions. In [8], an optimal channel estimation scheme based on a minimum variance unbiased estimator was proposed that provides the optimal design of the IRS phase shift matrix. In [9], the authors proposed a cascaded channel estimation for a large, intelligent meta surface-assisted massive multiple-input-multiple-output (MIMO) system. In [10], two simple channel estimation methods for IRS-assisted MIMO systems based on parallel factors (PARAFAC) tensor modeling were proposed. The authors of [11] proposed an efficient CSI estimation method for an IRS-assisted mm-wave network. In [12], the authors discussed the channel modeling and estimation for MIMO mm-wave communication systems using an on-off strategy that sequentially estimates the channel coefficients by turning on each IRS element one by one while keeping others off.

[13] explores the potential of IRS to enhance wireless sensing by leveraging low-cost passive elements. They discusses three IRS-aided sensing schemes: passive, semi-passive, and active sensing, comparing them in terms of performance, cost, and complexity. They also addresses key design challenges such as IRS deployment, channel acquisition, and reflection design. Numerical results demonstrate improved sensing accuracy with IRS, particularly highlighting the superior performance of IRS active sensing compared to other methods. [14] proposes a CSI-free scheme for massive wireless energy transfer using IRS to address channel estimation challenges due to the lack of active RF chains. It maximizes received energy through phased beam rotation while considering the effects of imperfect IRS elements. Qin et. al introduces an IRS-assisted orthogonal time frequency space (OTFS) modulation scheme for high-mobility scenarios, jointly optimizing OTFS frame structure and IRS phase shifts to enhance signal coherence. They employs location-aided channel estimation at the IRS and a delay/shifted-Doppler approach at the base station, paired with a low-complexity IIC detector, outperforming traditional methods in simulations [15].

Ohyama et al.[1] proposed an IRS system that can autonomously update the phase shift in order to improve both communication and localization without any external feedback. Intricate hardware design, however, restricts the scalability. Yan et al.[2] investigated IRS-assisted multicast beamforming under discrete phase shifts and showed significant performance improvement but suffered from the dynamic channel state. Sadia et al. [3] considered IRS-assisted non-orthogonal multiple access (NOMA) systems for throughput maximization. Although their approach achieved good results, it required high computational resources, which makes it infeasible for large-scale deployments. Ashraf et al. [16] introduced feedback-based beam steering mechanisms for metasurfaces, which significantly improves the beam alignment. However, their dependence on full CSI restricted the applicability of the method in fast-changing environments. More recent works have focused on low-complexity approaches, such as the work by Su et al. [17], which proposed efficient phase adjustments using heuristic algorithms. However, these methods often compromise optimality for simplicity.

Optimization of beamforming in IRS-assisted systems has also been widely investigated. Early works focused on the joint optimization of transmit and reflective beamforming to improve the system performance. For example, algorithms were developed to maximize the achievable rate by jointly designing the beamforming vectors at the BS and the phase shifts at the IRS, under the knowledge of only statistical CSI [18]. More recently, to further reduce the dependency on instantaneous CSI, machine learning techniques have been considered. Supervised learning models can be trained to predict optimal phase configurations from historical data, which allows real-time adaptation without explicit channel estimation. These approaches have been shown to achieve near-optimal performance with lower computational complexity [19]. Also, the combination of IRS with moving platforms has been considered to further increase coverage and flexibility. Dynamic optimization algorithms are developed to jointly optimize the position and phase shifts of movable IRS under the non-convex optimization problem and show better system performance in different scenarios [20].

A practical phase shift model addressing amplitude variations was proposed by Abeywickrama et al. [21], demonstrating significant performance gains through joint transmit and reflective beamforming optimization. However, the model’s computational complexity limits its applicability in real-time systems. Wu and Zhang [22] designed algorithms for discrete phase shift optimization in IRS-assisted networks, where the transmit power is minimized under SINR constraints. Although the achievable power gain is comparable to that of continuous phase shifts, the approach in [22] is limited by the mixed-integer non-linear programming complexity. These studies have showcased significant progress in IRS optimization but have also highlighted persistent challenges, including computational complexity, feedback overhead, and practical implementation constraints. These challenges underscore the need for innovative solutions that strike a balance between performance and scalability in IRS-assisted wireless communication systems.

While the majority of existing research focuses on explicit channel coefficient estimation, such methods often demand substantial training overhead and estimation time, which limits their practicality. To overcome these limitations, we propose two feedback-based algorithms that enable efficient and optimal phase adjustment of IRS elements without requiring explicit knowledge of channel coefficients at the transmitter. The first proposed algorithm employs a phase shift matrix (PSM)-based approach, where the IRS phase shift vector is intelligently selected from a predefined matrix to maximize the output SNR at the receiver, minimizing feedback overhead. The second algorithm introduces a single-bit feedback (SBF)-based phase adjustment method, where the transmitter iteratively makes small, random phase modifications to IRS elements. The receiver then sends a one-bit feedback indicating whether the SNR improved or deteriorated, allowing the transmitter to retain favorable adjustments while discarding unfavorable ones.

The key contributions of this study are summarized as follows:

- Design of a PSM-based algorithm that selects optimal phase shift vectors to maximize SNR with minimal feedback overhead.
- Introduction of a SBF algorithm to iteratively adjust IRS phases using a simple feedback mechanism, achieving near-optimal performance with low complexity.
- Evaluation of the performance trade-offs between the two algorithms, demonstrating the superior bit error rate (BER) performance of the SBF algorithm compared to the PSM-based approach.
- Demonstration of scalability, showing reduced iteration requirements as the number of IRS elements increases, validating the practicality of the proposed algorithms in large-scale deployments.

## System model

**Fig 1:**
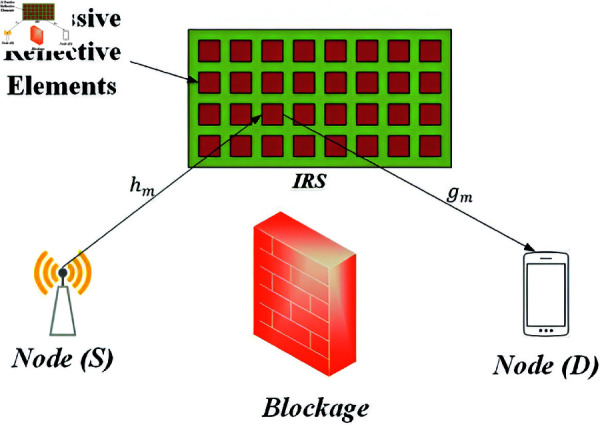
IRS-assisted system model.

Here, we assume an IRS-assisted single-input-single-output (SISO) wireless communication system in which a source node *S* communicates with a destination node *D* via *M* passive reflective elements, as shown in Fig 1. Both nodes are equipped with a single antenna. It is assumed that the direct link between *S* and *D* does not exist due to a severe scattering environment. Let *h*_*m*_ be the fading coefficient between *S* and ${\text{m}}^{\text{th}}$ reflective element, and *g*_*m*_ be the fading coefficient between the ${\text{m}}^{\text{th}}$ reflective element and *D*. Both channel coefficients are assumed to be circularly symmetric complex Gaussian distributed with zero mean and having variances ${\sigma_{h}}_{m}^{2}$, ${\sigma_{g}}_{m}^{2}$ respectively i.e., $h_{m}\sim 𝒞𝒩(0,{\sigma_{h}}_{m}^{2})$ and $g_{m}\sim 𝒞𝒩(0,{\sigma_{h}}_{m}^{2})$. Without channel estimation at *S*, phase adjustment of each IRS reflective element can be entirely configured by *S* to cater to the phase rotations introduced by *g*_*m*_ and *h*_*m*_ via efficient feedback algorithms.

**Fig 2:**
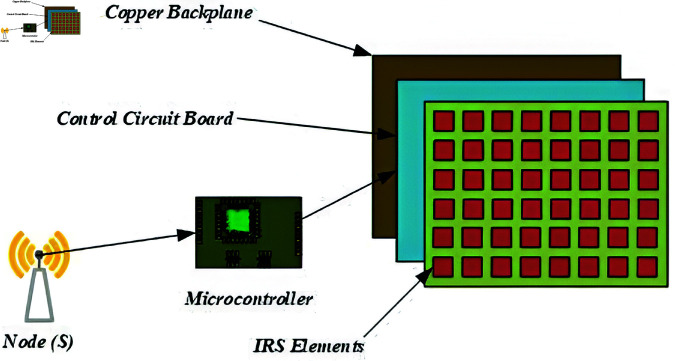
Generalized architecture of an IRS system.

Fig 2 shows the generalized architecture of an IRS system, which comprises the following key components:

- Metasurface Reflective Elements: The IRS consists of a thin, programmable metasurface composed of sub-wavelength unit cells, each capable of independently adjusting its reflection phase and/or amplitude.
- Control Circuit Board and Smart Controller: A dedicated low-power microcontroller unit (MCU) or a field-programmable gate array (FPGA) dynamically configures the phase shifts via pre-stored lookup tables or real-time learning-based approaches. The control signals are generated based on CSI feedback or pre-optimized IRS configurations.
- Copper Backplane and Isolation Mechanism: A metallic ground plane is integrated to prevent undesired signal leakage and backscattering, ensuring almost all incident energy is redirected toward the desired direction.

During the ${\text{k}}^{\text{th}}$ iteration, the received signal at *D* transmitted by *S* after passing through IRS is

$$y(k)=\sum_{m=1}^{M}g_{m}\Gamma_{m}e^{j\phi_{m}}h_{m}x(k)+n_{D}(k),$$ (1)

where *x*(*k*) is the information symbol transmitted by *S* with $𝔼\{|x(k){|}^{2}\}=1$, *n*_*D*_(*k*) is circularly symmetric complex additive white Gaussian noise (AWGN) at the *D* with zero mean and variance $\sigma_{n}^{2}$, i.e., $n_{D}(k)\sim 𝒞𝒩(0,\sigma_{nD}^{2})$. $\Gamma_{m}$ and $\phi_{m}$ denote the magnitude and phase of the reflection coefficient corresponding to the ${\text{m}}^{\text{th}}$ IRS element, respectively.

## Proposed phase adjustment feedback algorithms

In this section, we propose two novel feedback algorithms that can make efficient phase adjustments of IRS elements without explicit knowledge of CSI at the transmitter. These algorithms are designed to reduce feedback overhead and computational complexity while ensuring efficient IRS-assisted wireless communication.

### Phase Shift Matrix (PSM) based phase adjustment

In this algorithm, the IRS phase shift vector is selected from the available PSM to maximize the output SNR at the receiver, and the receiver only sends back the index corresponding to the best phase shift vector to the transmitter. More explicitly, at every certain period, which can be determined by the channel coherence time, the receiver feedbacks the index of optimum phase shift vector using $B=\log_{2}(N)$ bits, where *N* is the number of phase shift vectors in the PSM [23]. Then, the received SNR at *D* is

$$\gamma (k)=\rho {\left|\sum_{m=1}^{M}g_{m}\Gamma_{m}e^{j\phi_{m}}h_{m}\right|}^{2},$$ (2)

where $\rho =\frac{1}{\sigma_{nD}^{2}}$ is the average transmit SNR. Since the index of the optimum phase shift vector is chosen to give the maximum SNR, the optimum received SNR is

$$\gamma_{max}=\max\{\gamma_{1},\gamma_{2},\cdots ,\gamma_{N}\}\;=\rho \max_{1\leq l\leq N}{\left|\sum_{m=1}^{M}g_{m}\Gamma_{m}e^{j\phi_{m}(l)}h_{m}\right|}^{2}.$$ (3)

This maximization ensures the constructive addition of signal components, leading to the highest possible SNR at the receiver.

At the receiver, an optimum phase shift vector from the available PSM is chosen based on the maximum SNR, and its index is sent to the transmitter via the feedback channel. With long repetition codes’ availability, we assume there is no feedback error. Therefore, the transmitter will be able to configure phases of IRS elements correctly based on receiver feedback. This results in exact information on the channel fading coefficient not being required at the transmitter, which reduces the complexity of IRS-assisted wireless communication systems. This approach also significantly reduces the feedback overhead compared to traditional CSI-based methods, as only the index is transmitted. Moreover, the feedback complexity is logarithmic concerning the number of phase shift vectors, ensuring scalability.

### Single-Bit Feedback (SBF) based phase adjustment

In this algorithm, the transmitter makes a minor random alteration in the phase of each IRS element in each iteration; simultaneously, the receiver also transmits a SBF indicating whether the SNR has improved or deteriorated after the current iteration. The transmitter keeps the “good” phase adjustments and throws the “bad” ones [24]. The transmission is assumed to be noiseless, which can quickly be assured using large amounts of redundancy if needed. This binary feedback drastically reduces feedback overhead, making the algorithm highly efficient in practical systems.

The transmitter keeps a record of $\phi_{best,m}(k)$ of the best-known value of phase shift for each IRS element, where *k* is the iteration index. The receiver measures the received SNR γ(k) at each iteration *k* and keeps a record of the best received SNR $\gamma_{best}$ of its previously observed SNR, and is given by

$$\gamma_{best}=\max_{n\leq k}\gamma (n),$$ (4)

where

$$\gamma (n)=\rho {\left|\sum_{m=1}^{M}g_{m}\Gamma_{m}e^{j\phi_{m}(n)}h_{m}\right|}^{2}.$$ (5)

At the next iteration, *k* + 1, the transmitter makes a random phase disruption $\varepsilon_{m}$ from some probability distribution $f_{\varepsilon }(\varepsilon_{m})$ (this distribution can change over time). The updated phase shift is given by

$$\phi_{m}(k+1)=\phi_{best,m}(k)+\varepsilon_{m}.$$ (6)

The receiver then measures the received SNR and generates an SBF set to “1” if the received SNR in the current iteration is better than the previous best SNR and “0” otherwise. The receiver then broadcasts this feedback to the transmitter (the transmission is assumed to be error-free, which can be guaranteed using large numbers of redundancy if needed). The receiver upgrades its value of $\gamma_{best}(k\;+\;1)$ and the transmitter upgrades the phase rotations $\phi_{best,m}(k\;+\;1)$ to maintain the disruption $\varepsilon_{m}$ if the feedback bit is “1”, and throw them away otherwise. The process is repeated in the next iteration until the targeted received SNR is achieved. The updated process can be written mathematically as

$$\gamma_{best}(k+1)=\left\{ \begin{array}{cc} \gamma (k+1), & \gamma (k+1)>\gamma_{best}(k)\\ \gamma (k), & \text{otherwise} \end{array},\right.$$ (7)

and

$$\phi_{best,m}(k+1)=\left\{ \begin{array}{cc} \phi_{best,m}(k)+\varepsilon_{m}(k), & \gamma (k+1)>\gamma_{best}(k)\\ \phi_{best,m}(k), & \text{otherwise} \end{array}.\right.$$ (8)

This reinforcement learning-like approach ensures gradual convergence towards the optimal phase configuration. The feedback overhead is minimal, requiring only one bit per iteration, making it highly suitable for low-latency applications. However, the convergence speed depends on the choice of the perturbation distribution, which can be optimized for different scenarios in future work.

## Simulation results and discussion

A model of an IRS-assisted SISO system is implemented in MATLAB to test the performance of both the proposed algorithms to adjust the phases of IRS elements. Moreover, the relative performance of algorithms is evaluated. For all simulations, we took $\sigma_{h_{m}}^{2}=\sigma_{g_{m}}^{2}=$ –6 dB. BPSK is considered a modulation scheme without loss of generality.

**Fig 3:**
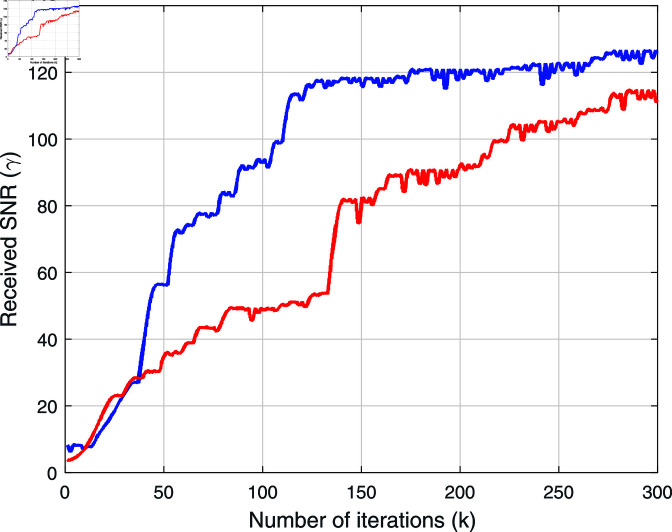
Two simulated instances with *M* = 16.

Fig 3 shows two simulated illustrations of the SBF algorithm, and we can see that the convergence of the two illustrations is close to each other. For any initial value of the phase shift $\phi_{m}$, the algorithm converges to perfect coherence almost surely. This convergence demonstrates the robustness of the SBF algorithm in maintaining signal coherence despite initial phase settings, a critical factor for practical deployment scenarios where initial conditions can vary widely.

**Fig 4:**
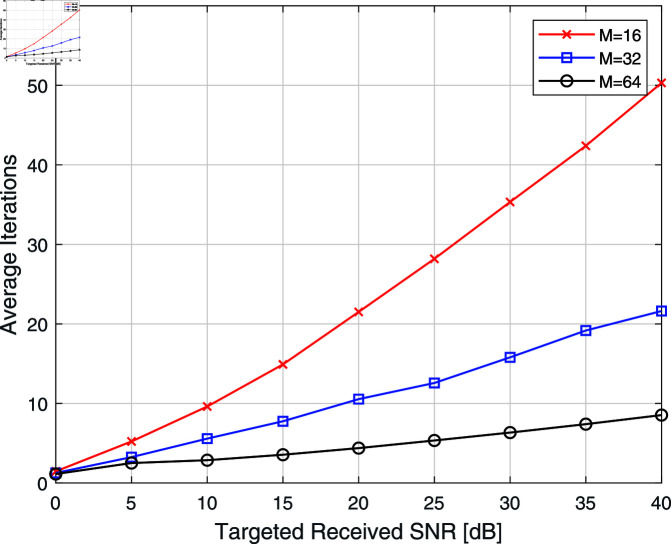
Different simulated instances with different values of *M.*

Fig 4 shows an average number of iterations against the targeted received SNR for different values of IRS elements *M*; it can be observed that the average iterations are a decreasing function of the *M*, i.e., the number of iterations required to achieve the same targeted received SNR for large value of *M* is less. The reason is, as the number of IRS elements *M* increases , received SNR γ also increases, and hence, fewer iterations are required to achieve targeted received SNR. This trend indicates that as the number of IRS elements increases, the system’s ability to manipulate the signal phase improves, leading to faster convergence rates. This scalability is vital for practical applications where large IRS arrays may be employed.

**Fig 5:**
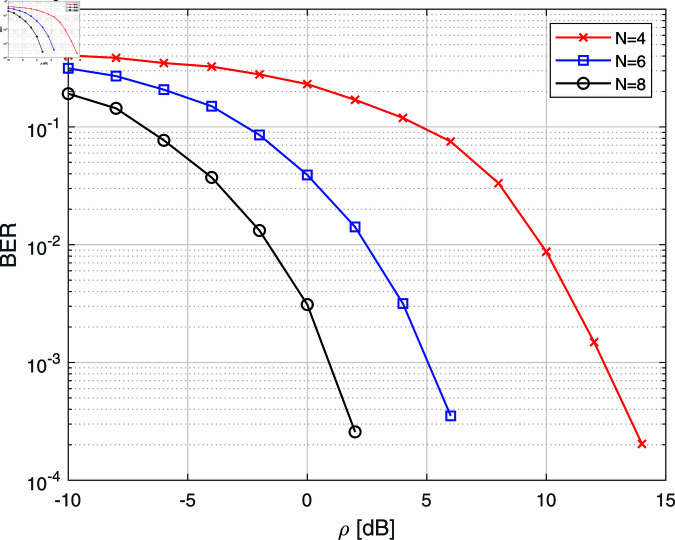
BER curve using Monte-Carlo simulation for *M* = 4 with different codebook lengths.

Fig 5 shows the performance of the average bit error rate (BER) of the system using *M* = 4, and PSM are given by $\phi_{1}=(\begin{array}{cc} -2.2494 & 0.9041\\ -2.7642 & -2.0961 \end{array})$, $\phi_{2}=(\begin{array}{cccc} 3.1416 & 0.7854 & -0.7854 & 0\\ 0 & -0.7854 & 0.7854 & 3.1416 \end{array})$ respectively. Notably, the BER performance improves as the PSM size expands, thus indicating that a larger phase shift matrix provides a finer granularity of phase control, which in turn enhances the signal alignment at the receiver. Therefore, increasing the feedback bits (*B*) can achieve better phase adjustment of IRS elements.

**Fig 6:**
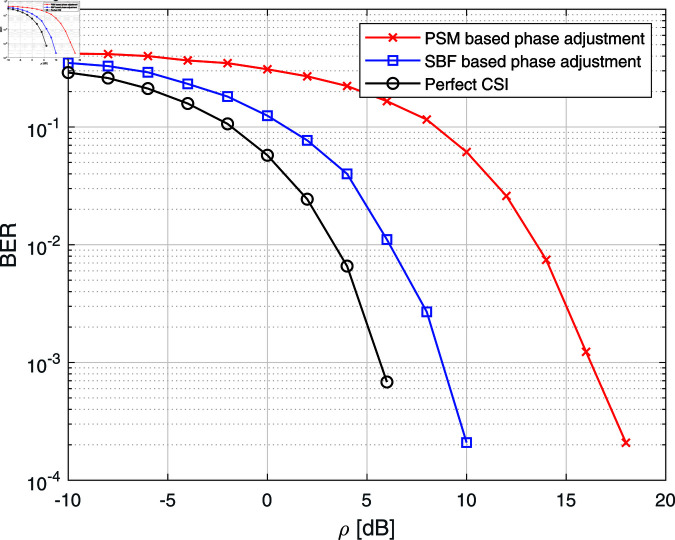
BER comparison among PSM-based feedback algorithm, SBF algorithm, and perfect CSI using Monte-Carlo simulation.

Fig 6 shows the performance comparison between the simulation results of the average BER of the system for the PSM-based feedback algorithm and SBF algorithm. This figure shows that the average BER performance of the SBF algorithm is very close to the actual CSI case. Because this algorithm iteratively produces phases by which the best phase adjustment (very close to the actual CSI case, i.e., $\phi ≃-(∡h_{m}\;+\;∡g_{m}$)) of IRS elements can be achieved. Therefore, the performance of the SBF algorithm outperforms the performance of the PSM-based feedback algorithm, highlighting its efficiency. However, it is important to note that the SBF algorithm is iteration-based and may require more time to converge, which could be a limitation in dynamic environments where channel conditions change rapidly.

In conclusion, the detailed exploration of each algorithm’s performance under varying conditions and configurations provides valuable insights into their practical applications and limitations. These insights are crucial for further refinement and implementation in real-world scenarios.

## Conclusions

In this paper, we considered the problem of phase adjustments of IRS elements for an IRS-assisted SISO wireless communication system. Two feedback-based algorithms (PSF and SBF) are proposed to adjust the phases of IRS elements; one is a PSM-based feedback algorithm, and the other is an SBF algorithm. In the PSM-based feedback algorithm, we produced the average BER curve using PSM size (*N* = 4, 6, and 8), and it is shown that the average BER performance can be improved as we increase the PSM size. It is also pointed out that since the PSM indexing can be determined offline, the calculation of our proposed PSM indexing does not affect the complexity of the IRS system. Next, it was shown that IRS phase adjustment using the SBF algorithm can be successfully implemented by employing only one bit of feedback per iteration from the receiver, and it is observed that its BER performance outperforms the PSM-based phase adjustment, but at the cost of convergence time. Both algorithms eliminate the need for explicit CSI at the transmitter, significantly reducing system complexity. Future extensions will be focused on systems consisting of IRS-assisted multiple-input-multiple-output (MIMO) wireless communication systems and choice of the perturbation distribution in SBF algorithm.
